# “You feel a bit unsexy sometimes”: The psychosocial impact of a spinal cord injury on sexual function and sexual satisfaction

**DOI:** 10.1038/s41393-022-00858-y

**Published:** 2022-10-13

**Authors:** Olivia E. C. Barrett, Emily Mattacola, Katherine A. Finlay

**Affiliations:** 1 School of Psychology and Clinical Language Sciences, University of Reading, Reading, Berkshire UK; 2 School of Psychology, The University of Buckingham, Buckingham, Buckinghamshire UK

**Keywords:** Rehabilitation, Rehabilitation

## Abstract

**Study design:**

A qualitative, semi-structured interview design.

**Objectives:**

This study aimed to identify, from the perspective of people living with a Spinal Cord Injury (SCI), the primary psychosocial barriers and facilitators that impact on their sexual function and sexual satisfaction post-injury.

**Setting:**

Community-dwelling sample of people with SCI in England, United Kingdom

**Methods:**

Semi-structured interviews with twenty people with SCI (15 males; 5 females) were conducted using an 8-item interview schedule. Inductive thematic analysis was undertaken of verbatim transcripts coded using Braun and Clarke’s (2021) six phases of thematic analysis.

**Results:**

Six inductive themes were generated, collectively describing the psychosocial barriers and facilitators impacting on sexual function and satisfaction post-SCI: (1) Internalising societal views and stigmatisation; (2) Diminished sexual confidence; (3) Navigating communication; (4) Managing relationship dynamics; (5) Lack of sexual support provision; and (6) Intervention development recommendations.

**Conclusion:**

Sexual function and satisfaction are highly challenging areas of rehabilitation for males and females living with SCI. Increased efforts are needed to educate others in society to overcome the negative stereotypical attitudes obstructing acceptance of sex despite disability. Countering sexual stigmatisation for people with SCI would facilitate growth in sexual confidence. Techniques to enhance interpersonal sexual communication and involve the partner/spouse in regaining mutual sexual satisfaction are foundational. The current study highlighted key outpatient-based recommendations for intervention development, clarifying primary targets for future SCI-focused sexual therapeutic work.

## Introduction

The World Health Organisation identifies sexual health as a fundamental human right for all individuals [1], yet people living with a disability often face exclusion from active sexuality and inaccurate assumptions of asexuality [2]. Though sexual activity can be considered integral to returning to satisfactory function after injury or illness [3], is it often an area of limited attention in rehabilitation [4]. Low sexual satisfaction rates are seen in older adults with long-term SCI [5], yet re-engaging with sexual activity contributes towards greater overall quality of life for spinal cord injured individuals [6]. Indeed, people who are able to adapt to their injury and maintain a satisfying sex life report better overall quality of life and life satisfaction [7]. Sexual function should therefore be a primary consideration for individuals living long-term with spinal cord injury [8]. Despite the evident importance of engagement with sex post-injury [9], sexual health and satisfaction continue to be under-addressed for this population [8, 10].

Part of the challenge of improving sexual function and satisfaction after SCI relates to the difficulty of providing and integrating adequate resources and education into rehabilitation [11]. Quantitative research has considered the physiological implications, barriers and complexities that impact on sexual functioning following injury [8, 12, 13]. However, qualitative research is more limited, demonstrating only that inadequacies in sexual support services can result in people with SCI experiencing reluctance and awkwardness in initiating discussion about this sex, limiting their requests for assistance [14–17].

Distinguishing the barriers and facilitators of sexual activity and satisfaction for people who are not currently in rehabilitation facilities and linking these with community-led recommendations for intervention development would provide a foundation for enhanced service provision. It is vitally important that individuals have accessible support and targeted psychoeducational provision around sexuality post-injury, including during the inpatient and outpatient rehabilitation phase. This qualitative research study therefore aimed to explore community-based perceptions of barriers and facilitators impacting sexual activity and satisfaction post-SCI.

## Methods

### Design

A qualitative semi-structured interview design was used, analysed via Thematic Analysis [18]. Semi-structured interviews allow interviewees to discuss sensitive subject areas in ways which feels accessible to them by discussing what they feel is important, without undue influence from others [19]. This study was pre-registered with Research Registry as part of a series of qualitative research studies investigating sexual function and satisfaction after SCI (Registration no. 6979).

### Participants

A purposive sampling technique [20] was adopted to identify individuals with SCI living in the community through Spinal Injury Case Management Ltd., a private case management company, UK-based, specialising in SCI care. To be eligible for inclusion, participants had to: (i) have a diagnosis of SCI according to the International Standard for Neurological Classification of Spinal Cord Injury (ISNCSCI); (ii) be aged over 18 years; (iii) have been living with SCI in the community for 18+ months; and (iv) have verbal proficiency in English. People with SCI who were currently undergoing treatment for either cancers affecting the spinal cord or a clinically diagnosed mental health condition were excluded. Twenty participants (5 females; 15 males) were recruited for this study, aged between 25 and 65 years (M = 49.95 SD = 13.42). No incentive was offered for participation. Participant demographics are presented in Table 1.

**Table 1:** Participant characteristics.

Pseudonym	Age	Gender	Sexual orientation	NLI/Completeness	Date of SCI	Current relationship status	Sex pre-injury	Sex post-injury
Elizabeth	27	F	Heterosexual	T4 complete	2015	Civil Partnership	Yes	Yes
Luke	57	M	Heterosexual	C4 incomplete	2014	Single	Yes	No
Robert	58	M	Heterosexual	C5 incomplete	2016	Married	Yes	No
Jack	31	M	Heterosexual	C4 complete	2014	Civil Partnership	Yes	Yes
Tom	33	M	Heterosexual	L2/3 incomplete	2015	Civil Partnership	Yes	Yes
Daniel	25	M	Heterosexual	T4 complete	2016	Engaged/Cohabiting	Yes	Yes
Beth	65	F	Heterosexual	T4 incomplete	2018	Married	Yes	No
Kate	28	F	Heterosexual	L4/5 incomplete	2018	Civil Partnership	Yes	Yes
Liam	59	M	Heterosexual	T10 incomplete	2017	Married	Yes	No
Ben	47	M	Heterosexual	T6 complete	2018	Married	Yes	No
Matthew	57	M	Bisexual	C6 complete	1982	Married	Yes	Yes
Jacob	64	M	Heterosexual	C4 complete	1974	Divorced	Yes	Yes
Nathan	62	M	Heterosexual	T12 complete	1985	Single	Yes	Yes
Mike	58	M	Heterosexual	T6 incomplete	1984	Married	Yes	Yes
Charles	53	M	Heterosexual	C5 complete	1985	Married	Yes	Yes
Billy	61	M	Heterosexual	T12/L1 incomplete	1979	Married	Yes	Yes
Jess	59	F	Heterosexual	T3 incomplete	2016	Cohabiting	Yes	No
Paul	53	M	Heterosexual	C6/7 complete	1985	Married	Yes	Yes
Lucy	55	F	Heterosexual	T5/6 incomplete	2016	Married	Yes	Yes
Oliver	47	M	Heterosexual	C5/6 incomplete	1988	Married	No	Yes

*SCI* spinal cord injury, *NLI* neurological level of injury.

### Materials

An 8-item semi-structured interview schedule was used to explore barriers and facilitators associated with post-rehabilitation sexual function and satisfaction in people with SCI (see Table 2). The interview schedule was developed through consultation of existing psychosexual literature which identified key areas for consideration as media influences [21], personal autonomy [11], and communication and relationship dynamics [22].

**Table 2:** Interview schedule.

1. What are your thoughts about how sex is portrayed by the media?
2. How important would you say sex and intimacy are in your life?
3. What impacts your *decisions* about whether or not you engage in sexual activity?
4. How have your levels of sexual satisfaction and desire been impacted following your injury?
5. What do you think the biggest challenges are for you in your sex life?
6. Some individuals feel happy and confident initiating discussions on sex whilst others find this quite difficult. How do you find communicating and talking about sex?
7. Having a spinal cord injury can sometimes change the dynamics of partner relationships which can impact on sex. What are your thoughts about and personal experiences of this?
8. How do you think sexual health management could be better addressed for individuals living with a spinal cord injury upon discharge into the community?

### Procedure

Prospective participants were invited via email lists maintained through the case management company, with information sheets sent out to eligible participants expressing an interest in the study. The consent form and demographics questionnaire were completed and returned electronically in advance of interview. Semi-structured interviews were conducted face-to-face (*n* = 4) or online using a virtual platform (*n* = 16). Participants were informed that their participation was voluntary, confidential and that the interview could be stopped/paused at any point. Interviews were audio-recorded and varied between 30 and 120 minutes (M = 65.15; SD = 26.29) in length, meeting guidelines for depth in qualitative research [23].

### Data analysis

An initial sample of 10 participants was set for recruitment [24], with a stopping criterion of three used to determine data saturation [25]. During analysis, no new information was generated after interview seventeen and no further themes emerged. The stopping criterion was tested for each subsequent interview (*n* = 3) until data saturation was confirmed.

Demographic data were summarized using descriptive statistics. Interviews were transcribed verbatim and then analyzed following Braun & Clarke’s (2021) six-step process; (1) Familiarization of the data; (2) Generation of initial codes; (3) Searching for themes; (4) Reviewing themes; (5) Naming the themes; and (6) Producing the report. Initial codes, representing individual barriers and facilitators were grouped together to develop higher-order themes. Inter-rater reliability analyses were performed in accordance with a coding reliability approach [26], which recommends the use of coding reliability in addition to thematic analysis to enhance applicability to clinical practice and rigour by triangulating codes between first and last authors. Miles & Huberman’s (1994) inter-rater reliability formula was used to confirm agreement, with the number of agreements divided by the total number of agreements plus disagreements, generating a high inter-rater agreement rate of 91%. Disagreements were resolved through discussion between the research team and in close consultation with field notes and interview transcripts.

### Ethical considerations

The University of Reading School of Psychology Ethics committee approved the research study. (Approval number: PWEC2020/47 P). Written informed consent was obtained prior to interviews and participants were informed about their right to withdraw, during and up to 48-hours after participation without giving a reason. The British Psychological Society Code of Human Research Ethics (2021) was adhered to throughout the research.

## Results

**Fig. 1:**
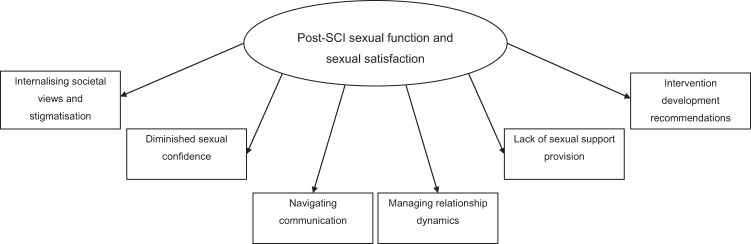
Qualitative Themes Identified by Thematic Analysis. The oval demonstrates the overarching superordinate thematic context of the results. The rectangles illustrate the six qualitative inductive themes evident in the data.

Six themes were identified from inductive thematic analysis, representing barriers and facilitators related to sexual function and satisfaction post-SCI: (1) Internalising societal views and stigmatisation; (2) Diminished sexual confidence; (3) Navigating communication; (4) Managing relationship dynamics; (5) Lack of sexual support provision; and (6) Intervention development recommendations (see Fig. 1).

### Internalising societal views and stigmatisation

Participants acknowledged that they faced a major barrier as a result of result of stereotypes, societal beliefs and stigmatisation pertaining to sexuality. Sexuality whilst living with a disability was felt to be under-addressed and misunderstood both within the media and wider society.

Sexuality amongst the disabled was perceived to be ignored by the media, increasing feelings of stigmatization and separation between people considered ‘normal’ and those living with a SCI:

A lot of the stuff in the media is relating to normal people. Well, I say normal; I mean people who don’t have a disability and people who don’t live in chairs, like able-bodied people if you know what I mean. I suppose I wouldn’t really class myself in the same situation as this anymore. (Jack, complete tetraplegia)

For many, media influences created negative pressure by normalising ableist sexual imagery that was disconnected from their personal reality and experience. This impacted on levels of self-esteem and sense of attractiveness:

I think it may make you feel a bit unsexy sometimes when you look at all those skinny models and supermodels. Like when you see an advertisement for Ann Summers and all of the women advertising the outfits are tiny with lovely figures. [It] doesn’t make you feel great about yourself really, does it? (Kate, incomplete paraplegia)

### Diminished sexual confidence

Rebuilding one’s confidence was difficult after SCI and elicited an array of emotions, including feelings of rejection, guilt and uncertainty, which collectively negatively impacted on sexual confidence. Formative setbacks to sexual confidence were particularly memorable during the early stages of re-engagement with sexual activity:

I remember being incredibly nervous the first time I engaged in real sexual activity after my injury. I was a first-year student at university and didn’t really know what to expect at all. I knew that was able to achieve a sustainable erection, but I also knew I was unable to ejaculate. (Oliver, incomplete tetraplegia)

Moreover, reduced body confidence and lowered self-esteem due to SCI-related body changes impacted on sexual and intimate activity and perceived attractiveness:

I had body issues hugely which were even worse before my suprapubic catheter. I put on three stone in weight and got a para-belly. Whilst I feel better after losing the weight in clothes, I don’t feel better in myself out of clothes. So, it has been quite frustrating all round really (Jess, incomplete paraplegia)

Managing diminished sexual confidence often necessitated a change in perspective, moving from an emphasis on penetrative sex to encompass intimate activity more broadly:

Over the years, I’ve learnt to enjoy sex in a much wider context of the word. Foreplay, touching and being touched and just holding somebody is just as important, if not more so than the simple act of intercourse which can sometimes be overrated and comes and goes in a flash. (Oliver, incomplete tetraplegia)

### Navigating communication

The need for communication around sexual function post-injury in partner, professional and social capacities was of central importance and was thought to facilitate sexual satisfaction. Effective communication aided in the initiation of open discussion, which helped to develop, establish and sustain a sexually healthy relationship:

I think to have that openness is really important and it helps to cement that part of the relationship. It is when things start going wrong and you hide things that it *all* starts to go wrong. Honestly, openness is the most important thing to sustain a healthy sexual relationship. (Mike, incomplete paraplegia)

Communication acted as a barrier for those entering and establishing new relationships: “I would be nervous about having a discussion with a partner or potential partner as you don’t know how someone will react, or more importantly what the consequences will be following that discussion” (Jacob, complete tetraplegia). Participants also highlighted difficulties communicating about sex due to the fear of being judged, given that, for many, sexual function is a sensitive and confidential life domain: “I think I would be a bit reluctant to just start discussing ‘personal’ matters with someone I didn’t know” (Beth, incomplete paraplegia).

### Managing relationship dynamics

In the process of adjusting to life with SCI, partner involvement was a vital component of support and acted both as a barrier and facilitator. Worries over partner satisfaction and the impact of one’s injury on the partner were highly salient. Physical changes and obstacles in relation to sexual functioning and performance led many individuals to question whether they were able to fulfil partner needs:

I feel that my partner misses out, but she said she isn’t bothered. Is she missing out? Well, I don’t truthfully know. She says not, but she isn’t going to say she is because she knows that will hurt me too. (Robert, incomplete tetraplegia)

However, there was a prominent shift from the focus on one’s own sexual satisfaction, to instead ensuring the sexual needs and satisfaction of one’s partner were met:

I probably get more out of the satisfaction I give to her as opposed to myself. I would sooner see her with a big smile on her face rather than myself. I mean some people are selfish and want it all to themselves, but you do start to satisfy the needs of your partner more, for certain. (Mike, incomplete paraplegia)

Distinguishing care roles, reducing the caregiving impact on one’s partner and, principally, finding a balance between giving and receiving sexual satisfaction helped to maintain a sexually healthy relationship. Care provision by the partner was thought to have the potential to differentially alter relationship dynamics and opportunity for sexual connection:

So now, he has to wash me down when I have been incontinent, he has had to empty urine bags and give me enemas, all that sort of thing. So, it completely changes the way he looks at me, even though he says it doesn’t. (Jess, incomplete paraplegia)

Physical assistance provided ahead of sexual engagement by the partner was initially perceived to be ‘off-putting’, but partner reassurance limited the negative impact of this on the relationship:

The hardest thing for me is my wife having to get me on the bed first, like help me on the bed, help me get undressed and all of that. Sometimes I felt it was a bit of a put off for her, but she said it isn’t at all. (Charles, complete tetraplegia)

Consequently, attempting to separate carer from partner roles was central to retaining relational stability.

### Lack of sexual support provision

All participants highlighted the lack of sexual rehabilitative services both within the inpatient and outpatient phases of recovery. The absence of educational provision and information was found to impact on sexual preparedness and reduce feelings of sexual competence and cognitive understanding. Healthcare professionals were considered important figures in the delivery and provision of education and support, yet approachability, confidence around clinical competence, accessibility and after-care upon discharge all were barriers.

Following fruitless attempts to search for answers and information, many became disillusioned:

You try to search for anything for women after a spinal cord injury and there isn’t anything. I mean I have been told numerous times, “you have lost your function and that’s it, now you just have to accept that”. It was never discussed with me in the spinal unit. (Jess, incomplete paraplegia)

For many, upon discharge from rehabilitation, there was a complete lack of knowledge around sexual function. This left the majority doubtful, uncertain and confused, a mental state exacerbated by difficulties approaching the spinal unit for further support upon discharge:

I find it hard how you are promised things and as soon as you get out of the hospital, you are no longer their problem when you phone back up. Once you are out and discharged, you are no longer their issue. (Charles, complete tetraplegia)

There was a clear reluctance to and avoidance of initiating discussions around sex with medical professionals. Most felt that clinicians also felt awkward and uncomfortable broaching the subject. The need for further information, discussion and support was collectively emphasised:

I know it’s easy to say it, but I think sexual health, sexual function and fertility should all be better discussed and covered as part of one’s rehabilitation after a spinal-cord injury…we need to get better at realising that sexual function and sexual relationships etc is part of everyday life and people’s needs and wants should be discussed more. (Oliver, incomplete tetraplegia)

### Intervention recommendations

Individuals varied in terms of their readiness to explore sex, yet, collectively agreed that some form of increased sexual education should initially be offered during the inpatient rehabilitative phase with further readily accessible after discharge into the community. Acknowledgement of differences in needs was referenced and, given the diversity of individual requirements, a group intervention format was felt to be inappropriate:

The set-up was completely wrong, and it was males and females together. I know one-to-one might be more time consuming when you’re in hospital which is why they work the bowels and bladder workshops around groups, but for sex I just don’t think groups is the answer. (Robert, incomplete tetraplegia)

It was felt that accessible, educational support and information was a facilitator, particularly if one could access it, across both inpatient and outpatient phases of recovery. Participants felt that the development of targeted reading materials should be prioritised to maintain privacy and promote advice-seeking:

The good thing about a book, like sexual function made easy for spinal cord injury, that’s more likely to get attention that anything else. It is private too and then people could seek advice if they wanted to. (Billy, incomplete paraplegia).

## Discussion

The current study aimed to understand the barriers and facilitators that may challenge sexual function and satisfaction post-spinal cord injury. The results demonstrated the emergence of six themes: *Internalising societal views and stigmatisation; Diminished sexual confidence; Navigating communication; Managing relationship dynamics; Lack of sexual support provision* and *Intervention recommendations*. The importance of sex and intimacy in pre-injury lives was collectively experienced as playing an important part in overall rehabilitation, consistent with wider findings [8, 12, 14]. Barriers due to societal beliefs, stigmatisation and the negative media portrayal of sexuality and disability meant people with SCI felt they were viewed as different to able-bodied ‘others’. Participants felt that those living with a disability were perceived as asexual or sexually incapable, impacting sexual interaction and interpersonal communication about sex by people with SCI and their Healthcare Professionals [21]. Working to better educate and overcome societal ignorance and disability-related myths may increase relationship and sexual opportunities for those living with a SCI.

The current study identified that reconciling, through open communication, the impact of involvement of one’s partner in SCI care duties and their (separate) role in sexual activity was paramount. Resolving the interplay between these roles was felt to help maintain relationship stability and positively impact on sexual function and intimacy, as established in research more widely [22]. This study demonstrated that a key barrier was concern about one’s ability to provide sexual fulfilment for one’s partner. Ensuring partner satisfaction was discussed and prioritised from both a sexual and intimate perspective, helped overcome and compensate for the impact of caring [27].

Changes in the nature of the relationship due to care-giving responsibilities have been found to lead to emotional and interpersonal challenges [28], as validated by this study. *Managing relationship dynamics* demonstrated that alterations in relationship dynamics were associated with physical care requirements. Role change from spouse/partner to caregiver was a key component in the fluctuation in relationship dynamics, causing strain and pressure [28]. Valuing sex, intimacy, or both, featured as a key component in sustaining a healthy relationship, with partner support playing a vital role in helping to overcome obstacles associated with sex and intimacy post-injury.

The importance of communication around sexual function was highlighted in personal, professional, and social contexts. Effective communication with the partner facilitated initiation of open discussion about sexual satisfaction and function. Those living with SCI report higher levels of sexual satisfaction when in an enduring relationship [29], thus it is important for therapeutic support efforts to be directed towards overcoming barriers to interpersonal, partner-to-partner communication. Broaching sexual function with healthcare professionals additionally proved problematic at times due to feelings of embarrassment and the fear of judgement, therefore identifying healthcare professionals with whom individuals feel most comfortable discussing sexual matters may help to overcome sexual function communication avoidance. Supporting people with SCI, their partners, and healthcare professionals to adopt strategies which promote effective communication about sex and which direct and support discussion, may help overcome such barriers, enhancing opportunities for enhanced sexual satisfaction.

Diminished body and sexual confidence presented a barrier to sexual function and satisfaction. Poor self-esteem and a post-injury changes in body image have resulted in feelings of sexual incapableness and mind-body disconnectedness [30], echoing findings in this study whereby reduced body esteem, self-esteem, perceived status of attraction and lack of spontaneity interfered with levels of sexual confidence, and positive evaluations of sexual and intimate activity. Further research suggests that consideration of the impact of weight gain, body scarring and changes in physical appearance may cause individuals to shield themselves from future sexual activity and self-exposure [31]. Consequently, equipping people with SCI with helpful strategies to better address reduced sexual confidence and body image issues would help reduce the magnitude of these psychosocial barriers. Additionally, support around continence management, for example, in preparation ahead of and during sexual activity should be taught [31] as it was consistently referenced as a barrier to perceived attractiveness in this study. Psychosexual education specific to people with SCI would be a strong candidate for future intervention development, as there is typically a positive relationship between sexual education and sexual activity [11].

Finally, in *intervention recommendations*, it was felt that there was a need to re-evaluate the level of current support available around sexual function post-injury. It was clear that proposed timings for intervention delivery were not limited to the inpatient rehabilitation phase, with outpatient support also required [9, 11]. The inclusion of the partner is considered a key facilitator, and group-based interventions were considered an inappropriate set-up due to sensitivity and embarrassment around the subject of sexual function. Recommendations were made that targeted written materials could be developed as an effective tool to promote increased knowledge and awareness of support for sexual function and satisfaction.

A number of limitations were evident during this study. Firstly, the gender imbalance in the study sample. However, this reflects the male-centric distribution of people with SCI in the UK, and so the sample is representative of the broader population. Secondly, those who took part had completed their SCI inpatient rehabilitation in spinal units across England, and therefore their experiences may not replicate the experiences of those who have completed SCI rehabilitation elsewhere. Future research could aim to explore sexual function and satisfaction cross-culturally and may also aim to recruit people with broader sexual preferences, including members of the LGBTQ + population.

This study explored the psychosocial barriers and facilitators related to sexual function and satisfaction following SCI. The findings demonstrate that stigmatizing stereotypes, dysfunctional communication, changes in partner role due to care needs, weak continuity of sexual support services after discharge from inpatient care, and diminished body confidence all act as barriers. Conversely, partner involvement, one-to-one intervention delivery, and the development of open communication about sex and mutually valuing of intimacy acted as facilitators. This research provides a qualitative baseline from which future interventions could consider developing tailored psychosexual interventions to improve sexual satisfaction and function post-injury.

## Data Availability

The data sets generated and/or analysed during the current study are available from the corresponding author on reasonable request, under institutional restrictions.
